# Reverse Hierarchical Processing of Speech in Talker Identification

**DOI:** 10.1111/ejn.70520

**Published:** 2026-04-22

**Authors:** Ja Young Choi, Shengyue Xiong, Jacie R. McHaney, Bharath Chandrasekaran

**Affiliations:** ^1^ Department of Communication Sciences and Disorders Northwestern University Evanston Illinois USA

**Keywords:** human, pupillometry, speech, voice

## Abstract

Spoken language contains critical information related to “what” is being said as well as “who” is talking. Listeners use various cues from speech to identify talkers. Talkers can be identified primarily based on acoustic cues, but access to higher‐level cues depends on the extent of language familiarity. Do listeners have greater processing costs when there is a greater number of cues available? Or do listeners leverage cues in a reverse hierarchical manner, using higher‐level cues more strategically to reduce processing costs? Here, we leveraged multiple methodological approaches, including pupillometry, analysis of the behavioral error pattern, and drift diffusion models (DDMs), to examine the dynamics of how learners identify novel talkers and accrue sensory evidence, balance cue usage, and manage processing costs during talker identity learning. Native English‐speaking adults learned to identify talkers in English and Mandarin, then were tested on recognizing them speaking new sentences, all while their pupillary responses, a proxy for processing cost, were recorded. Talker identification was more accurate and induced less pupil dilation in English relative to Mandarin. Analysis of error patterns showed that listeners relied more heavily on low‐level acoustic features in Mandarin than English, and DDMs revealed that higher evidence accumulation rates were associated with smaller pupil dilation. These findings suggest that, whenever available, listeners primarily use higher‐level abstract representations to identify talkers from the beginning of the learning process while relying on less efficient, lower‐level features of speech sounds in unfamiliar languages, consistent with the predictions of the reverse hierarchical framework.

## Graphical abstract

Beyond replicating the well‐established language familiarity effect—better talker identification in the native (English) versus unfamiliar (Mandarin) language—we incorporated drift‐diffusion model, error pattern analysis, and pupillometry to understand the time course dynamics of talker learning. Even early in learning, native‐language identification showed reduced pupil‐indexed effort, less reliance on between‐talker acoustic similarity, and higher evidence accumulation rates. The persistent gap between languages supports reverse‐hierarchy theory: learners bypass lower‐level representations when higher‐level, abstract representations are available.

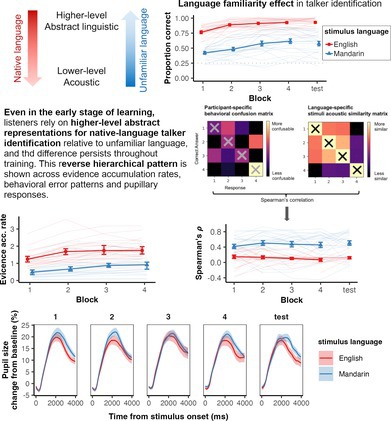

**Abbreviations**
ANOVA: analysis of variance
ASM: acoustic similarity matrix
DDM: drift diffusion model
GCA: growth curve analysis
HL: hearing level
ITI: intertrial interval
LFE: language familiarity effect
RHT: reverse hierarchy theory
RMS: root mean square
SPL: sound pressure level

## 1 Introduction

Human speech conveys both linguistic content (what is being said) and indexical information (who is speaking). Listeners are remarkably efficient at extracting both types of information simultaneously from the speech signal; they can recognize talkers from nonlinguistic content and comprehend linguistic content despite talker variability. However, this does not imply that the “what” and “who” information in speech are entirely dissociable. On the contrary, they are interdependent. Variability across talkers imposes processing costs on speech perception (Choi et al. 2018; Mullennix and Pisoni 1990), and talker identification is less accurate when the speaker uses an unfamiliar language. Native listeners thus have an advantage in identifying talkers, a phenomenon known as the language familiarity effect (LFE; Goggin et al. 1991; Thompson 1987).

Prior work has used multiple behavioral approaches to reach a consistent conclusion that talkers are more easily identified in a familiar language. Voice lineup studies demonstrated that people were better at identifying a voice from a set of voices in a familiar language than in an unfamiliar language (e.g., Goggin et al. 1991; Thompson 1987; Köster and Schiller 1997) and that people identified voices better when they spoke a familiar accent (other‐accent effect; e.g., Stevenage et al. 2012; Yu et al. 2021). Training studies, which track learning over time, similarly found LFE (Orena et al. 2015; McLaughlin et al. 2019; Perrachione and Wong 2007; Xie and Myers 2015; Bregman and Creel 2014). Through these studies, LFE has been robustly established across a range of languages including English, Spanish, French, German, Korean, and Mandarin.

What levels of linguistic representation drive the LFE when we learn new voices? Some studies have shown that phonological familiarity alone is sufficient or at least the primary factor that drives the effect of language familiarity (Fleming et al. 2014; Zarate et al. 2015; Yu et al. 2021). However, emerging evidence points to higher‐level lexical and semantic cues as key contributors of LFE (Bregman and Creel 2014; Xie and Myers 2015). Collectively, these findings from previous studies suggest that (i) while talker identification is possible to some extent in the absence of linguistic comprehension, listeners preferentially leverage higher‐level linguistic representations whenever accessible, rather than relying solely on acoustic cues of talkers, and that (ii) the use of higher‐level cues makes talker identification more accurate.

Nevertheless, behavioral findings alone provide limited insight into the time course and dynamics of talker learning. Here, we revisit LFE using a combination of physiological measures and behavioral modeling to understand how cue usage and processing costs in talker learning evolve over time. Such understanding can also allow us to adjudicate between contrastive models of perceptual learning that could scaffold talker learning: the reverse hierarchy theory (RHT; Ahissar and Hochstein 2004; Ahissar et al. 2009; Nahum et al. 2008) and the two‐stage perceptual learning model (Jiang et al. 2018; Serre et al. 2007).

RHT proposes that perceptual judgements are guided primarily by high‐level abstract representations unless access to those representations is blocked or attention is explicitly directed to lower‐level sensory features (Ahissar et al. 2009; Ahissar and Hochstein 2004; Nahum et al. 2008). In this view, talker learning in both native and non‐native languages is supported by the same underlying mechanism, with differences arising from the level of representation available early in learning. Within this framework, listeners have immediate access to higher‐level cues for native speech but not for non‐native language. By contrast, in the two‐stage perceptual model, learning begins with bottom‐up extraction of lower‐level features, which form the early scaffolding for later‐emerging, higher‐level categorical representations (Jiang et al. 2018; Serre et al. 2007). Thus, both frameworks posit that native and non‐native talker learning shares a common mechanism, but they differ in their accounts of whether learning begins from higher‐level abstract representations or from lower‐level sensory analysis.

Both models share the view that the representational level that drives learning shifts with expertise and context, but they make different predictions about the time course of perceptual learning. RHT would treat native‐ and non‐native‐language talker learning as outcomes of the same learning mechanism while making different predictions of early learning profiles depending on whether abstract representations are readily accessible: native‐language talker learning should be relatively efficient from the beginning, less effortful, and less dependent on bottom‐up acoustic cues, whereas unfamiliar‐language talker learning should initially be harder, more effortful, and more acoustically driven. With training, RHT predicts improvement in both languages but with a persistent gap in effort and acoustic cue reliance because higher‐level scaffolding remains limited in unfamiliar language. The two‐stage feedforward model, however, predicts a different trajectory of talker learning. Early learning is effortful in both native and unfamiliar languages because of the reliance on bottom‐up feature extraction; over time, talker identification in both languages will become more efficient and less effortful as more abstract category representations emerge, with differences between languages reflecting the rate of tuning rather than a qualitatively different strategy early in learning. While the two models do make different predictions about talker learning trajectory, we cannot adjudicate between the two models by using behavioral accuracy and response time measures alone.

In this study, we aimed to better understand the dynamics of LFE in talker learning by incorporating behavioral error patterns, pupillometry, and drift diffusion modeling (DDM). Native English listeners with no prior experience in Mandarin Chinese were trained to identify a set of native English‐speaking talkers and native Mandarin‐speaking talkers while their pupillary response was recorded.

Behavioral accuracy across listeners' native and non‐native languages is sufficient to establish LFE, but error patterns in the behavioral task can provide additional important insight into how listeners weight the usage of acoustic vs. higher‐level linguistic cues over the time course of talker learning. For example, by comparing the (dis)similarity patterns in talkers' low‐level acoustic features with the listeners' behavioral confusion patterns, Xiong et al. (2025) demonstrated that reliance on acoustic cues decreases not only with native‐language experience but also as the listener gets more familiar with the talker's accent. Using a similar approach as Xiong et al. (2025), we aimed to examine how acoustic cue reliance changes as the listeners learned to identify new talkers.

Pupillometry indexes processing cost and effort over the course of learning. Pupil dilation reflects arousal and effort during cognitive tasks (Beatty 1982) and provides a reliable marker of processing costs and the encoding and retrieval of task‐relevant information (Papesh et al. 2012). It has been widely used to assess cognitive load and listening effort across different levels of task difficulties (Winn et al. 2015; McHaney et al. 2021, 2024; McGarrigle et al. 2017; Borghini and Hazan 2018; Zekveld et al. 2013; Kuchinsky et al. 2016). Pupillary response also varies with subjective interpretations of stimuli, demonstrating the influence of higher‐level control factors (Naber and Nakayama 2013; Strauch et al. 2022). Because talker identification may recruit different processing levels depending on language familiarity, pupillometry can reveal how cue use shifts across languages and how those shifts affect processing cost.

DDMs take both trial accuracies and response times to model decisional processes during perceptual tasks (Ratcliff 1978; Ratcliff and McKoon 2008; Smith and Vickers 1988), allowing us to integrate these measures in the context of speed‐accuracy trade‐off. DDMs assume that the decision‐maker accumulates evidence for multiple possible decision options at varying rates (evidence accumulation rate), and that a decision is made when the accumulated evidence reaches a particular threshold for one of the options (decision threshold). Thus, within the framework of DDM, the evidence accumulation rate reflects the efficiency with which a decision‐maker attends to task‐relevant features of the stimuli (Kiani et al. 2008; Pereira et al. 2021; Smith and Vickers 1988). In the context of our study, this parameter is particularly relevant as we are interested not only in how quickly or accurately listeners identify talkers, but also in the specific cues they use to identify talkers, and how different cue usage patterns contribute to their effectiveness in talker identification. Because listeners can rely on cues of differing efficiency (more efficient higher‐level vs. less efficient lower‐level cues) depending on language familiarity, a higher evidence accumulation rate would imply greater reliance on higher‐level, abstract representations. DDM has not only been successful at describing various aspects of human perception and behavior (reviewed in Ratcliff et al. 2016), but also relates to baseline and phasic pupil dilation (de Gee et al. 2014; Dix and Li 2020; McHaney et al. 2024). The association between pupil dilation and decisional parameters is likely reflective of shared neural mechanisms involving the locus coeruleus‐norepinephrine system (Aston‐Jones and Cohen 2005; Gold and Shadlen 2000).

Taken together, our multimodal design allows us to quantify changes in cue use via error patterns, processing cost via pupillary response, and efficiency of information extraction via evidence accumulation rates. By tracking these measures over the course of talker learning in native and unfamiliar languages, we test whether early learning reflects immediate access to abstract representations (RHT) or initial bottom‐up feature extraction that subsequently tunes higher‐level mappings (two‐stage feedforward model). RHT predicts that, even in the early stage of learning, native‐language talker learning will show lower pupil‐indexed effort, higher evidence accumulation rate, and fewer acoustically driven confusions than unfamiliar‐language talker learning—and that these gaps will remain persistent throughout the course of training. In contrast, the two‐stage model predicts minimal early differences between languages in pupil‐indexed effort, evidence accumulation rate, and acoustic cue reliance; with training, both languages should exhibit reduced effort, increased evidence accumulation rate, and decreased reliance on acoustic cues, but the native language should show a steeper improvement rate, yielding growing gaps in the rate of change across measures.

## 2 Materials and Methods

### 2.1 Participants

24 Native English‐speaking adults were recruited (15 female, 9 male; ages 19–33 years; mean = 25.75 years, SD = 6.24 years). All participants were self‐reported native speakers of English with no history of language or hearing disorders and no prior experience with Mandarin Chinese. All participants passed the hearing screening test with pure tones of 250 Hz to 8 kHz hearing thresholds being lower than 25 dB HL. All participants gave written informed consent, and all study procedures were approved by the Institutional Review Board at Northwestern University.

### 2.2 Stimuli

We used audio recordings of 10 English sentences and 10 Mandarin sentences, spoken by 4 native English speakers and 4 native Mandarin speakers respectively (Bradlow, n.d.). Stimuli were normalized for RMS amplitude to 70 dB SPL in Praat (Boersma and Weenink 2023).

### 2.3 Task Procedure

The study was divided into two sessions separated by at least two hours, each of which contained one of the two stimulus languages—English or Mandarin. The order of the two languages was counterbalanced across participants. Auditory stimuli were presented through Sennheiser HD 280 Pro over‐ear headphones.

Pupillometry was recorded while participants completed a talker identification task. Participants' left eye pupil size was recorded at a sampling rate of 1000 Hz, using an Eyelink 1000 Plus Desktop Mount (SR Research; Ottawa, Ontario, Canada) with a chin and forehead rest for stabilization. The room lighting was set at a fixed, dim level for all participants to ensure that the luminance of the visual field was consistent. A nine‐point eye tracker calibration was performed before each language session started.

**FIGURE 1:**
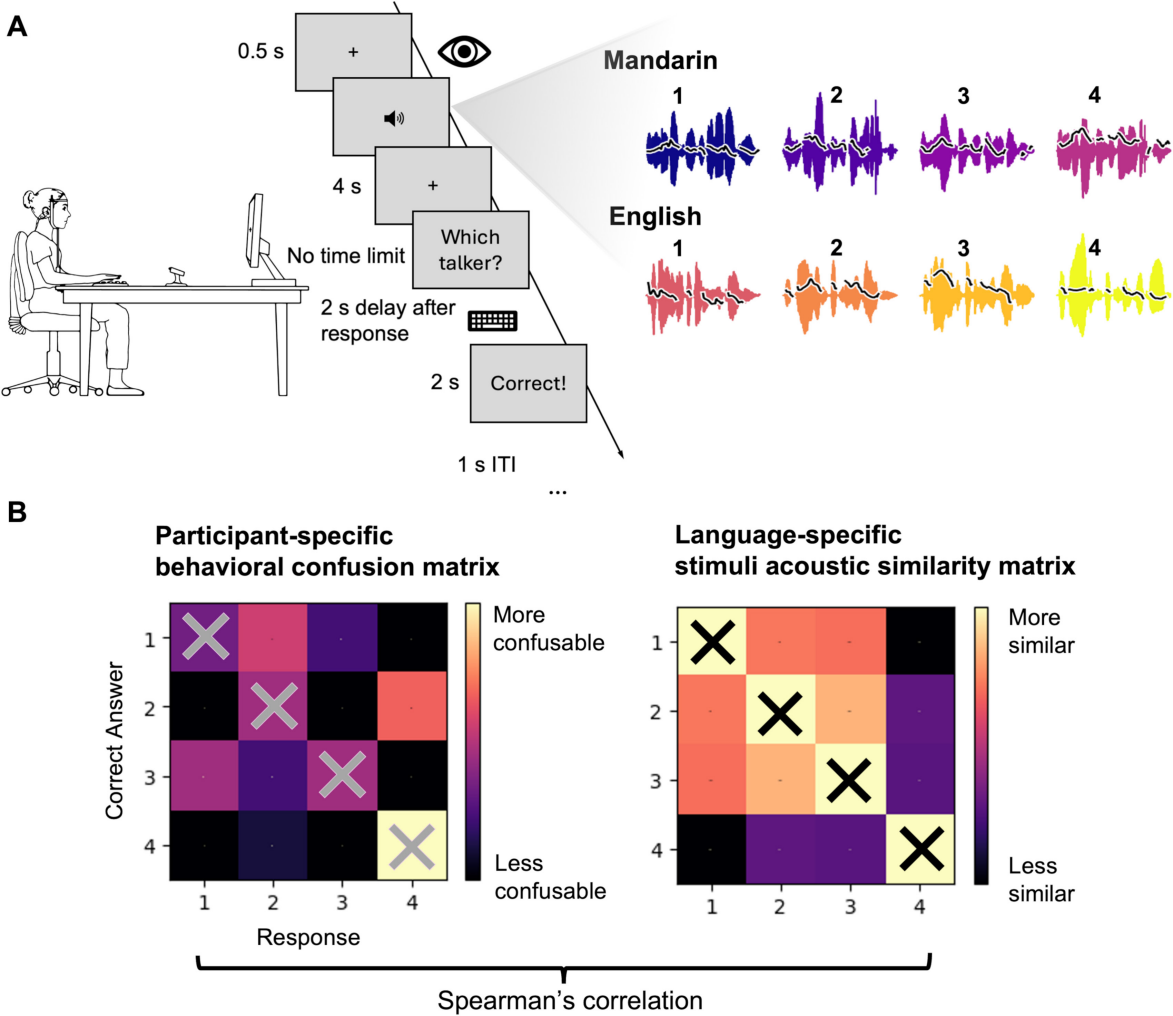
(A) Task procedure. Participants performed talker identification task in English and Mandarin, with a 4‐s delay between stimuli and response and a 2‐s ITI. Procedure shown in the figure is the structure of one trial in training phase; the corrective feedback and the 2‐s delay before the feedback were removed in test phase. (B) Error pattern analysis. The relationship between participants' error patterns and stimuli acoustic similarities were analyzed using Spearman's rank correlation.

Each language session was composed of a training phase and a test phase. The training phase was composed of four blocks, and the test phase was one block. In both phases, each block contained 40 trials. Because the pupillary response unfolds slowly over time, we included a delay after each trial event to allow for changes in pupil size that were not contaminated by subsequent trial events (Koelewijn et al. 2018; McHaney et al. 2021, 2023; Zekveld et al. 2013; Figure 1A). To control for the effects of saccades and minimize pupil foreshortening (Hayes and Petrov 2016; Knapen et al. 2016), participants fixated on a cross in the center of the screen for a minimum of 500 ms at the beginning of each trial. After this fixation criterion was met, the sentence stimulus was presented. There was a four‐second delay from the offset of the stimulus to the response prompt on the screen in the form of “Which speaker?”, and participants had unlimited time to respond by pressing the number key (1, 2, 3, or 4) that corresponds to each of the four talkers in the language. Following the response, there was a two‐second delay before corrective feedback was given in the form of the text “Correct” or “Incorrect” displayed on the screen for two seconds. After the feedback, there was an intertrial interval (ITI) of one second.

In all the four training blocks, participants heard the same 10 sentences spoken by the four talkers (40 total stimuli), with every sentence stimulus presented once per block. In the test phase, participants heard 10 novel sentences spoken by the same four talkers, with each stimulus presented once in the test block. The order of the auditory stimuli was randomized within each block.

Manual drift correction was performed between each block by the experimenter to ensure high quality tracking of the pupil. Between blocks, participants were given the option to take a short break to prevent fatigue. Pupillary data from the four‐second period starting from the onset of the stimulus for each trial were used for further analysis.

### 2.4 Data Analysis

#### 2.4.1 Behavioral Data Analysis

##### 2.4.1.1 Accuracy

Accuracy was coded as 1 for correct and 0 for incorrect responses for each trial. Data were analyzed in R (version 4.3.2) using generalized linear mixed‐effects models implemented in the package *lme4* (Bates et al. 2015). Separate models were estimated for the training phase and test phase. The model for the training phase included fixed factors of language, block and their interaction, random within‐participant slopes for language and block, and random intercepts for participants (Barr et al. 2013). The model for the test phase included a fixed factor of language, a random within‐participant slope for language, and random intercepts for participants. Contrast for the language factor was treatment‐coded with English as the reference level. Significance of fixed factors was determined in a Type III analysis of variance (ANOVA). Significant effects from the ANOVA were followed by testing specified contrasts on the terms in the linear mixed‐effects model. We adopted a significance criterion of α = 0.05, with *p* values in the mixed‐effects linear models based on the Satterthwaite approximation of the degrees of freedom using the *lmerTest* package (Kuznetsova et al. 2017).

##### 2.4.1.2 Response Time

Response times were measured from the onset of the response prompt being displayed on the screen and were log‐transformed to ensure normal distribution. Outlier trials deviating from each participant's mean log response time in each block by more than 3 standard deviations were excluded from the analysis (1.76% of total trials).

Data were analyzed using the same combination of software and packages as described in the section above (2.4.1.1), with the exception that linear mixed‐effects models were used instead of generalized linear mixed‐effects models. As done with accuracy data, separate models were estimated for training and test phases: the model for training phase included fixed factors of language and block while the model for text phase included the fixed factor of language only, and both models included random within‐participant slopes for language and random intercepts for participants.

#### 2.4.2 Drift Diffusion Modeling

We used the Bayesian implementation of the drift‐diffusion model (DDM) developed by Paulon et al. (2021) to compare the dynamics of the decision processes in the two language tasks (English, Mandarin) as a function of training. For every combination of response (*d*) and stimulus category (*s*), the DDM estimates an evidence accumulation rate parameter (*μ*_*d*,*s*_) and a decision threshold parameter (*b*_*d*,*s*_) that were fit. Evidence accumulation rate reflects how efficiently the decision‐maker extracts relevant information from the stimulus. Lower evidence accumulation rates are common for more difficult tasks when extracting relevant information from the stimulus is more challenging. The decision threshold parameter represents the amount of evidence that the decision‐maker needs to accumulate in order to make the decision, hence reflecting the decision‐maker's response caution (Bogacz et al. 2010). Higher decision thresholds suggest that the listener was more cautious to make a response, favouring accuracy over speed when making decisions. Importantly, this DDM allowed for the evidence accumulation rate and decision threshold parameters to vary between individuals, to control for the substantial variability across participants. The DDM also allowed for evidence accumulation rate and decision threshold parameters to change slowly over time across learning blocks. This helps to account for the changes in decision‐making processes during learning. Additionally, for each stimulus category (talker), an offset parameter was fit, which represents the time taken by stimulus encoding and motor response—actions that are not considered to be directly relevant to the decision process.

We estimated the DDM parameters separately for the two language conditions (English and Mandarin), as the participants came in for two separate sessions to complete the two language conditions. DDMs were run using the *lddmm* package in R (Paulon and Sarkar 2023). We used a Bayesian framework that assigned priors to the parameters and relied on samples drawn from the posterior using a Markov chain Monte Carlo algorithm for estimation and inference. Prior to estimating the DDMs, the top and bottom 1% of trials based on response times were excluded as outliers to improve the model fit. Markov‐chain Monte Carlo simulations were used to run 6000 iterations, with the initial 2000 samples discarded as burn‐in. The remaining iterations were thinned in intervals of 5 to reduce autocorrelation.

Consistent with previous auditory learning studies which showed that gradual improvement in correct categorical decisions characterizes learning (Roark et al. 2021, 2024), we focused on the DDM parameters associated with correct trials only (i.e., *μ*_*d*,*s*_ and *b*_*d*,*s*_ where *d* = *s*). Also, we included data only from the training phase and not the test phase because we were specifically interested in how the process of learning may impact the decisional strategies underlying talker learning in the two languages. We examined the posterior probabilities of evidence accumulation rate and decision threshold parameters for each language and each block. Posterior means are reported as point estimates and 95% pointwise credible intervals were used to assess uncertainty. Differences across blocks were inferred when the 95% pointwise credible intervals were nonoverlapping.

#### 2.4.3 Pupillometry Data Analysis

##### 2.4.3.1 Preprocessing

We analyzed pupillary data from −500 to 4000 ms from the onset of the speech stimulus. These pupillary data were preprocessed to remove noise (Winn et al. 2015; Zekveld and Kramer 2014). First, the data were downsampled to 50 Hz (Wierda et al. 2012), and any trial with more than 15% of its samples detected as blinks was removed (12% of total trials rejected; McMahon et al. 2016). Missing samples due to blinks and saccades were linearly interpolated to 120 ms before and after the blink. For each trial, the average pupil size in the 500 ms prior to the onset of the stimulus was used as the baseline pupil size, and the following pupil size after the stimulus onset was normalized against the baseline (Peysakhovich et al. 2015); the outcome variable reported in the *Results* section is the proportion change in pupil size relative to the baseline.

##### 2.4.3.2 Growth Curve Analysis

Pupil responses within the time window from 0 to 4000 ms time‐locked to the speech stimulus onset were averaged across trials for each block for each participant and were analyzed using growth curve analysis (GCA; Mirman 2014). We used GCA for analyzing the pupillary responses because it provides a statistical approach for modeling changes in the timing and shape of the pupillary response over time, which does not follow a linear trajectory (Winn et al. 2015, 2018).

A GCA was fit to model the interactions between first‐, second‐, third‐, and fourth‐order orthogonal polynomials and the independent variables of interest. The fourth‐order polynomial was chosen to model the pupillary response because it was consistent with the trajectory of the data and provided a better fit than a model that included only first‐, second‐, and third‐order polynomials. This fourth‐order model uses five parameters to map the trajectory of the pupillary response: the intercept reflects the overall change in the pupillary response over the entire time window and can be interpreted as the mean change in pupil size over time. The linear term (ot1) represents the slope of the pupillary response over time. If the linear term is positive, this reflects the rate of dilation, while a negative linear term reflects the rate of constriction. The quadratic term (ot2) reflects the curvature of the pupil response. A larger, negative quadratic term suggests a more parabolic shape and values closer to zero reflect a more linear shape. The cubic and quartic terms (ot3, ot4) reflect the extent to which secondary and tertiary inflection points occur in the pupil response, respectively (Kuchinsky et al. 2014; Mirman 2014). The absolute value of each term reflects the strength of the pupil response, while the polarity of the term reflects the direction of the response.

GCAs were conducted using the *lme4* package (Bates et al. 2015) with log‐likelihood maximization using the BOBYQA optimizer for convergence (Mirman 2014), and *p*‐values were estimated using the *lmerTest* package (Kuznetsova et al. 2017). We first estimated a GCA to examine the effect of language and training block. The model included fixed effects of each growth curve time term (ot1‐ot4), language, block, and all two‐ and three‐way interactions between time terms, language, and block. The random effect structure included a random intercept of participant and random slopes of participant on each time term.

We also estimated separate GCAs to examine how the DDM parameters of decision‐making were reflected in the pupillary response during listening. For this analysis, separate models were estimated for English and Mandarin conditions, only for trials that were categorized correctly. The models included fixed effects of the DDM parameter (evidence accumulation rate) on all time terms and all two‐way interactions between the DDM parameter and time terms. The GCAs also included a random intercept of participant and random slopes of participant on each time term.

#### 2.4.4 Error Pattern Analysis

We used an error pattern analysis method similar to the approach by Xiong et al. (2025). Behavioral confusion matrices were constructed for each participant, for each block in each language, such that each cell in the matrix represented the proportion of hearing a talker and identifying them as each talker (e.g., out of all the trials where the participant heard Talker 3, the proportion of identifying them as Talker 4). For the purpose of this analysis, we excluded the data on the diagonals of these matrices as we aimed to analyze the error patterns only.

Two separate acoustic similarity matrices (ASMs) were constructed for English talkers and Mandarin talkers. To compute the similarities, six different acoustic measures were taken into account: mean F0, range of F0, speech rate, jitter, harmonicity, and formant dispersion. These features were selected following a previous study on perceptual dissimilarity judgement of voices (Perrachione et al. 2019). Using these features, dissimilarity matrices were first computed based on the Euclidean distances between talkers using the Python *rsatoolbox* library (https://rsatoolbox.readthedocs.io). ASMs were created by multiplying −1 to the dissimilarity matrices.

To ensure that the acoustical variance was comparable between the English and Mandarin speech samples used in the study, we ran an analysis comparing the variance of speech samples between the two languages. A Mann–Whitney–Wilcoxon test on the Euclidean distances between talkers in each language showed that there was no significant difference in acoustical variance of the speech stimuli between the two languages (*W* = 17, *p* > 0.93).

After the behavioral confusion matrices and the stimuli ASMs were constructed, we ran a correlational analysis between them using Spearman's rank correlation (Figure 1B). The Spearman's correlation coefficients were compared between the two language conditions. Stronger positive correlations would indicate that the error patterns follow acoustic similarities of the voices more closely.

## 3 Results

### 3.1 Behavioral Results

#### 3.1.1 More Accurate Talker Identification in English Than Mandarin

**FIGURE 2:**
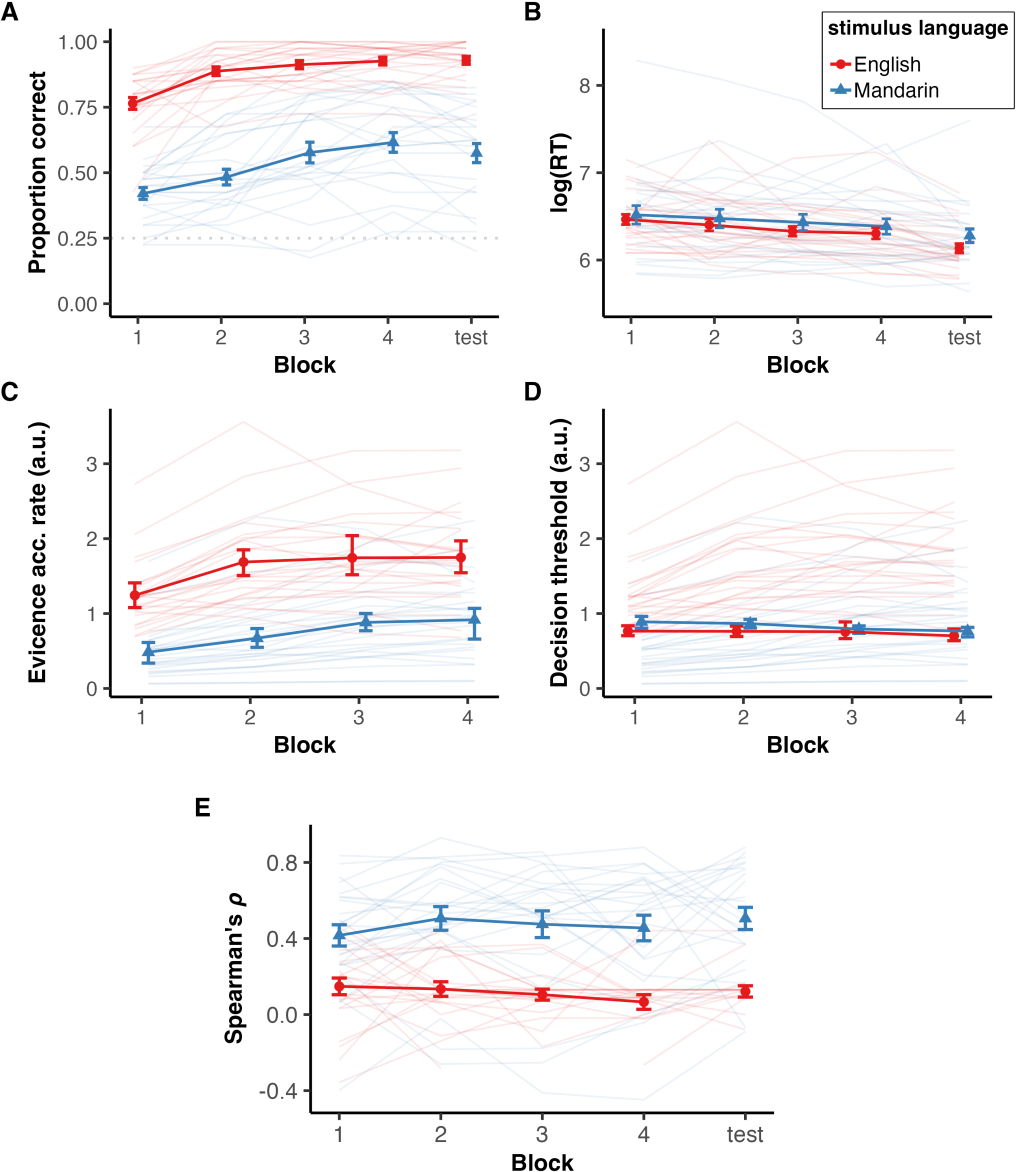
(A) Accuracies and (B) log (response time (ms)) of talker identification in each block in each language. Points and error bars indicate mean ± SEM. (C) Evidence accumulation rates and (D) decision thresholds estimated for drift diffusion models, in arbitrary units (a.u.). Points and error bars indicate mean ±95% credible intervals. (E) Mean ± SEM Spearman's correlation rank coefficient between each participant's behavioral confusion matrix and stimuli acoustic similarity matrix in each language. Higher coefficient indicates higher reliance on acoustic features when identifying talkers. In all panels, thin, semi‐transparent lines show individual participant data.

We examined whether talker identification accuracy was different in the English and Mandarin conditions and whether it improved over the blocks in the training phase (Figure 2A). There was a significant main effect of block (*χ* ^2^ = 44.78, *p* < 0.001) showing that participants learned to identify talkers over the course of training. We observed a significant main effect of language such that participants identified talkers in English more accurately than in Mandarin (*χ* ^2^ = 353.61, *p* < 0.001), replicating LFE shown by previous studies. We also observed a significant interaction between language and block (*χ* ^2^ = 23.55, *p* < 0.001), suggesting different magnitudes of learning across training blocks between the two languages. The specified contrast terms in the model revealed that the effect of block on Mandarin was smaller than on English (*β* = −0.29, *s.e*. = 0.059, *z* = −4.85, *p* < 0.001), showing that the effect of training was greater in English than Mandarin. The pattern of higher accuracy in English compared to Mandarin persisted in the test phase (*χ* ^2^ = 125.99, *p* < 0.001).

#### 3.1.2 No Llanguage Difference in Response Time

We observed a significant effect of block on response time in the training phase such that the response times became faster with more training (*χ* ^2^ = 13.56, *p* < 0.001; Figure 2B). There was no significant effect of language on response times in the training phase (*χ* ^2^ = 0.33, *p* = 0.56) or interaction between block and language (*χ* ^2^ = 0.98, *p* = 0.32). The response times were not significantly different between English and Mandarin in the test phase (*χ* ^2^ = 3.14, *p* = 0.076).

### 3.2 DDM Results

#### 3.2.1 Evidence Accumulation Rate Is Higher in English Than Mandarin

Overall, participants had higher evidence accumulation rates across all blocks for English sentences relative to Mandarin sentences. In English, the evidence accumulation rate increased from block 1 to block 2 and plateaued for the rest of the training phase (Figure 2C). For Mandarin, the evidence accumulation rate increased from block 1 to block 3 and plateaued afterwards. This suggests that listeners became more efficient at extracting relevant information from stimuli for decision‐making faster in English than in Mandarin.

#### 3.2.2 No Difference in Decision Thresholds Between Languages or Between Blocks

Decision thresholds remained stable throughout the training phase for both English and Mandarin and were not different between language conditions (Figure 2D). This indicates that listeners were just as cautious to make a response in the English condition as in the Mandarin condition.

### 3.3 Greater Reliance on Acoustic Features in Mandarin Than English

We analyzed the association between behavioral error patterns and the acoustic similarities between talkers' voices in order to assess the listeners' reliance on talker‐specific acoustic features when identifying talkers. We observed a stronger positive correlation between the behavioral error (e.g., likelihood of hearing Talker 1 and identifying them as Talker 3) and the acoustic similarity (e.g., how similar Talker 1 and Taker 3's acoustics are) in Mandarin than in English (Figure 2E). These results reveal that listeners' identification of talkers more closely matched the acoustic similarities between the talkers in the unknown language compared to the native language.

### 3.4 Pupillometry

#### 3.4.1 Greater Pupil Dilation in Mandarin Than English

**FIGURE 3:**
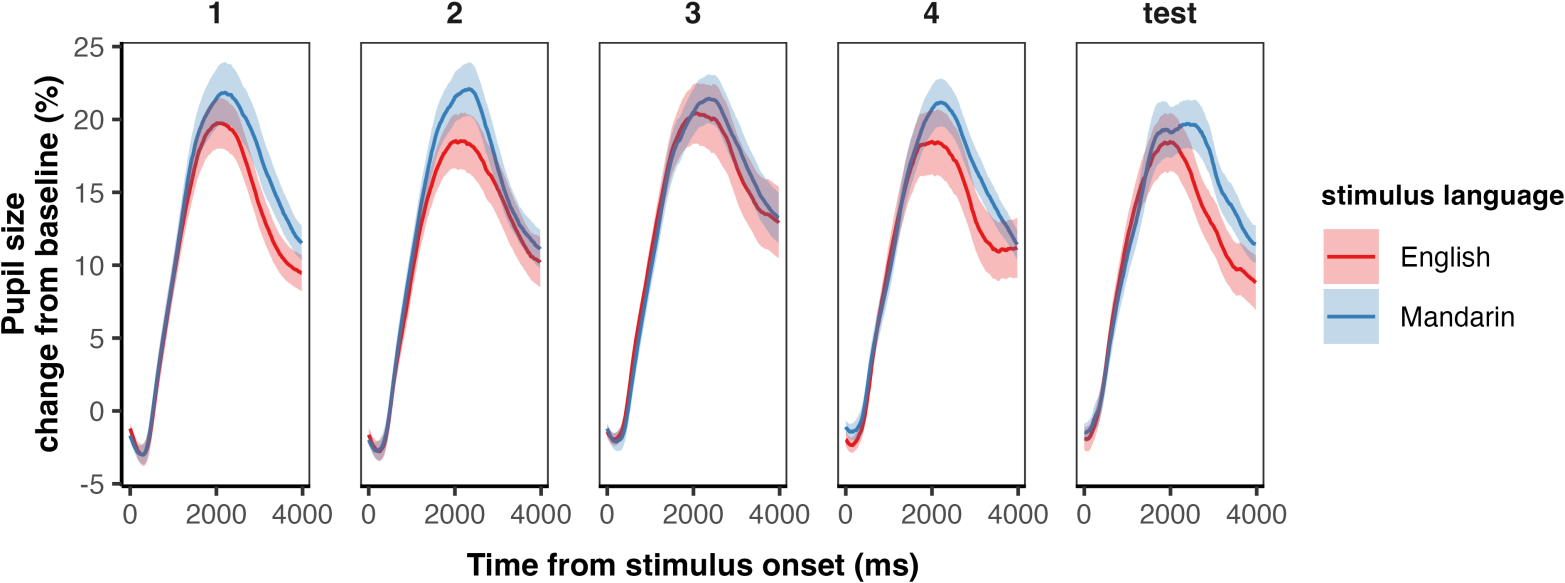
Changes in pupil dilation across time in each block, showing increased processing cost in Mandarin than English. Each participant's pupil diameter was normalized to their pre‐stimulus baseline pupil diameter. The shaded region indicates standard error.

The average pattern of pupillary response in each language for each block is illustrated in Figure 3. Growth curve analysis (GCA) showed a significant effect of language on overall pupil dilation in the first block of the training phase, with increased pupil diameter in Mandarin than in English (*p* < 0.001; Table 1). Linear and quadratic polynomial terms contributed significantly to the difference between the two languages (*p*s < 0.001), such that the rate of overall dilation was faster in Mandarin than English, and the primary curvature was stronger in Mandarin than English. There was no significant effect of language on secondary and tertiary curvatures of pupil dilation (*p*s > 0.60). These results reveal the effect of language on the pupil dilation during stimulus presentation rather than post‐stimulus processing, as the quadratic polynomial term is more representative of the initial increase of pupil diameter.

**TABLE 1:** Fixed effects estimated for growth curve models on pupillary responses to study the effect of language and block.^a^

Contrast	*β*	*s.e*.	*t*	*p*
(Intercept)	11.5	0.87	13.13	< 0.001***
ot1	5.67	6.34	8.94	< 0.001***
ot2	−82.3	5.12	−16.08	< 0.001***
ot3	−0.23	3.14	−0.072	0.94
ot4	24.5	2.36	10.39	< 0.001***
Language (Mandarin vs. English)	1.80	0.14	12.94	< 0.001***
ot1 × language	12.6	1.98	6.39	< 0.001***
ot2 × language	−8.56	1.98	−4.33	< 0.001***
ot3 × language	1.02	1.98	0.51	0.61
ot4 × language	0.64	1.98	0.32	0.75
block	0.19	0.036	5.31	< 0.001***
ot1 × block	−0.52	0.510	−1.01	0.31
ot2 × block	2.81	0.510	5.51	< 0.001***
ot3 × block	2.82	0.510	5.54	< 0.001***
ot4 × block	−1.47	0.510	−2.88	< 0.01**
language × block	−0.21	0.051	−4.05	< 0.001***
ot1 × language × block	−0.66	0.721	−0.91	0.36
ot2 × language × block	1.23	0.721	1.7	0.089
ot3 × language × block	−3.21	0.721	−4.45	< 0.001***
ot4 × language × block	0.0030	0.721	0.004	1.00
Test phase				
(Intercept)	11.5	1.21	9.44	< 0.001***
ot1	33.0	6.57	5.02	< 0.001***
ot2	−71.9	5.97	−12.04	< 0.001***
ot3	17.1	3.13	5.46	< 0.001***
ot4	14.5	2.58	5.62	< 0.001***
language (Mandarin vs. English)	1.61	0.078	20.61	< 0.001***
ot1 × language	22.7	1.11	20.50	< 0.001***
ot2 × language	−0.0049	1.11	−0.004	1.0
ot3 × language	−11.45	1.11	−13.11	< 0.001***
ot4 × language	−0.090	1.11	−0.81	0.42

^a^ The GCA model in R notation: lmer(pupil ~ (ot1 + ot2 + ot3 + ot4)*language*block + (ot1 + ot2 + ot3 + ot4 | participant), control = lmerControl(optimizer = “bobyqa”), data = gca.data, REML = FALSE).

^**^ *p* < 0.01.

^***^ *p* < 0.001.

There was a significant effect of block on the intercept, quadratic, cubic, and quartic polynomial terms in English (*p*s < 0.01), showing that more training led to a larger change in the pupillary response over time, flatter primary and tertiary inflection points, and a sharper secondary inflection point. There was also a significant interaction effect of language and block on the intercept and cubic polynomial term (*p* < 0.001), suggesting that over the course of the training, the effect of language difference on the overall pupillary response and the curvature at the peak of the pupillary response decreased.

#### 3.4.2 Greater Evidence Accumulation Rate Associated With Reduced Pupil Dilation

We examined the extent to which the decisional processes were reflected in the pupillary response by estimating GCAs within each language condition separately on average pupillary responses as a function of evidence accumulation rates (Table 2). In both English and Mandarin, a greater evidence accumulation rate was associated with reduced overall pupil dilation, a slower rate of dilation, and flatter primary and tertiary curvatures (*p*s < 0.01). Regardless of the type of evidence being accumulated in the two languages, more efficient extraction of evidence from the auditory stimuli resulted in reduced processing costs.

**TABLE 2:** Fixed effects estimated for growth curve models on pupillary responses fitted separately for English and Mandarin, to study the effect of evidence accumulation rates (EA Rates).^a^

Contrast	*β*	*s.e*.	*t*	*p*
English				
(Intercept)	15.61	1.01	15.50	< 0.001***
ot1	73.76	8.14	9.06	< 0.001***
ot2	−95.47	5.41	−17.64	< 0.001***
ot3	7.55	3.89	1.94	0.056
ot4	26.27	3.01	8.74	< 0.001***
EA Rate	−2.46	0.12	−19.11	< 0.001***
ot1 × EA Rate	−14.20	1.76	−8.04	< 0.001***
ot2 × EA Rate	13.19	1.68	7.86	< 0.001***
ot3 × EA Rate	0.47	1.58	0.30	0.77
ot4 × EA Rate	−43.80	1.39	−2.74	< 0.01**
Mandarin				
(Intercept)	13.64	1.16	11.80	< 0.001***
ot1	71.95	7.47	9.63	< 0.001***
ot2	−92.18	6.35	−14.52	< 0.001***
ot3	2.15	4.19	−0.51	0.61
ot4	26.15	3.08	8.49	< 0.001***
EA Rate	−0.66	0.15	−4.52	< 0.001***
ot1 × EA Rate	−12.81	2.05	−6.26	< 0.001***
ot2 × EA Rate	13.95	1.93	7.22	< 0.001***
ot3 × EA Rate	0.72	1.97	0.36	0.72
ot4 × EA Rate	−5.24	1.71	−3.06	< 0.01**

^a^ The GCA model for each {language} condition in R notation: lmer(pupil ~ (ot1 + ot2 + ot3 + ot4)*EA_Rate + (ot1 + ot2 + ot3 + ot4 | participant), control = lmerControl(optimizer = “bobyqa”), data = {language}.gca.ddm.data, REML = FALSE).

^**^ *p* < 0.01.

^***^ *p* < 0.001.

## 4 Discussion

We investigated the dynamics of talker identification by using two language contexts that varied the richness and types of speech cues available to the listener. Extending upon the traditional approach of measuring talker identification accuracies under different language conditions, we derived decisional processes using drift diffusion models (DDMs), analyzed the listeners' reliance on acoustic features of speech via behavior‐acoustic correlation analysis, and measured listeners' pupillary response as they completed the talker identification task. Consistent with prior work, listeners identified talkers more accurately in their native language than in a language they did not know. In the last training block and the test block, their accuracy was better than the initial block of training in both English and Mandarin, but the difference between the two languages was still significant. We observed an overall increase in pupil dilation when listeners were identifying talkers in a non‐native language compared to the native language, both during the training phase and the testing phase. Combined with the behavioral‐acoustic analyses, the differences in processing cost and accuracy between identifying talkers in a native versus non‐native language likely arise from listeners relying more heavily on bottom‐up acoustic cues when processing speech in a non‐native language, whereas they depend more on top‐down language‐based cues in their native language.

### 4.1 Reliance on Bottom‐Up Processing of Acoustic Features in Talker Identification in an Unfamiliar Language

Our analysis of acoustic measures of talkers' voices and participants' error patterns reveals that listeners rely more heavily on talker‐specific acoustics in an unfamiliar language than in their native language, replicating the previous finding (Xiong et al. 2025). As listeners have only limited phonological cues and no higher‐order linguistic knowledge in an unfamiliar language, they can only process the low‐level acoustic features of the talkers who speak an unfamiliar language. As a result, the behavioral error patterns were more closely associated with the acoustic similarities of the stimuli in listeners' unfamiliar language than in their native language.

Even at the very beginning of the training, listeners already showed a significant difference in acoustic cue reliance between native and unfamiliar languages. This reveals that, as soon as the higher‐level abstract representations were available (i.e., native language), listeners were relying on them instead of extracting lower‐level acoustic features, consistent with the predictions of RHT (Ahissar et al. 2009).

When listeners hear different talkers and judge how dissimilar the talkers are, their perceptual dissimilarity judgement does not differ among listeners with different native languages (Perrachione et al. 2019). This shows that listeners' native language experience does not affect perceptual sensitivity to talker acoustics, enabling them to process and compare the acoustic features of different talkers regardless of the language spoken. However, when asked to *learn to identify* the talkers rather than to *judge how dissimilar* the pairs of talkers are, listeners in our study drew from more than just the acoustics of the stimuli when higher‐level linguistic knowledge was available.

A potential limitation of this analysis is that there are fewer errors in English than Mandarin, thus making the range of error proportions in English smaller than in Mandarin. However, the fact that the behavior‐acoustic correlation in Mandarin remained strong even as the accuracies improved in the last training block shows that listeners consistently relied heavily on the acoustic features of the voices in a language that they do not understand. In the absence of top‐down guidance, learning did not reach beyond the bottom‐up processing of acoustic features.

### 4.2 Greater Pupil Dilation and Lower Evidence Accumulation Rate When Identifying Talkers in an Unfamiliar Language

We observed greater pupil dilation in the non‐native than the native language at the beginning of the training phase, and the pupil dilation difference between the two languages decreased as the training progressed through later blocks.

Not only did we observe increased pupil dilation associated with talker identification in the unfamiliar language relative to the native language, but we also observed that evidence accumulation rates in the unfamiliar language were lower than in the native language and did not increase beyond the third block. Rather than a gradual increase in evidence accumulation rate, which would be predicted by the two‐stage feedforward model, the increase in efficiency of evidence extraction in the unfamiliar language seemed to plateau. Listeners were able to access more abstract levels of speech in their native language, so they readily gained efficiency in accumulating relevant information for talker identification, and the gap between the two languages persisted, more consistent with the predictions of RHT.

Our observations are in line with previous neuroimaging studies on processing native vs. non‐native languages. Performing tasks in one's non‐native language has been associated with increased processing demands (Berken et al. 2015; Borghini and Hazan 2018), and identifying talkers in the native language produced weaker response relative to non‐native languages in the superior temporal cortex, supramarginal gyrus, and inferior frontal gyrus (Meng et al. 2024). However, greater proficiency with the non‐native language has been associated with recruitment of a more “classical” language network (Chee et al. 2001; Leonard et al. 2011) and more left‐lateralized brain regions, such that the neural recruitment for non‐native language processing resembles native language processing. In our study, the pupillary response differences between the two languages decreased as the listeners became more familiar with identifying talkers in the non‐native language. Future neuroimaging studies focusing on the dynamic talker learning trajectory will be able to further adjudicate between RHT and two‐stage feedforward model, as the two models make different predictions on the primary brain region that would support talker learning: RHT would predict that higher‐order language area such as inferior frontal gyrus would drive talker learning in the native language, whereas the auditory cortex would drive non‐native talker learning; two‐stage model would predict auditory cortex tuning before higher‐order representations emerge in the brain for both languages but with better representational fidelity for the native language.

Our analyses, integrating pupillometry data with evidence accumulation rates, revealed that a higher rate of evidence accumulation corresponded to smaller overall pupil dilation in both languages. This suggests that greater efficiency in extracting relevant information from stimuli leads to reduced cognitive processing demands, as reflected by decreased pupil dilation. Nonetheless, as discussed above, in their native language, listeners accumulated information in a way that enables higher‐level processing of the speech stimuli; in their unfamiliar language, listeners could only primarily gather information on the acoustic features of each talker. While these talker‐specific acoustic features may help with learning outcomes, processing more acoustic features and maintaining the information in working memory incur more processing costs than using more abstract representations, which is reflected in the pupillary response difference between native and unfamiliar languages. Taken together, our findings indicate that although the underlying mechanism for talker identification is consistent across native and unfamiliar languages, differences in the type and quality of evidence obtained in non‐native language impede the efficiency of evidence accumulation, thereby increasing the processing cost in non‐native language contexts.

A parsimonious interpretation of the results is that talker identification can be primarily carried out operating on the higher‐level, abstract representations of the speech stimuli, rather than low‐level sensory features, in line with reverse hierarchy theory (Ahissar and Hochstein 2004). Although previous studies have identified some key acoustic features that enable voice similarity judgements (Baumann and Belin 2010; Perrachione et al. 2019), learning to identify talkers based on their speech production inevitably involves processing of the linguistic information, as long as the listener is very familiar with the language. Language familiarity affords listeners the ability to perceive speech signals in relation to higher‐level, more abstract representations rather than granular processing of acoustic features. Primarily accessing the high‐level representations makes perception more efficient, whereas impaired ability to understand the complex relations between features leads to increased processing cost and more pupil dilation (Naber and Nakayama 2013). Thus, the pupil dilation increase in non‐native talker identification may reflect disrupted high‐level processing of the unfamiliar‐language speech stimuli. Taken together, the results reveal that talker identification in one's native language does not require the sequential processing of phonological, lexical, and semantic levels of speech. Instead, listeners can directly access abstract higher‐level cues, thus not incurring additional processing costs that would be predicted if the listeners were processing all different levels in a hierarchical, sequential manner. In fact, this ability to process higher‐level information in one's native language allows for a more efficient cognitive resource allocation by minimizing the processing of lower‐level acoustic features. While there has been evidence of brain regions and mechanisms that are specifically tuned to human voice processing (Belin et al. 2000; Latinus and Belin 2011), our findings demonstrate a strong interdependence between speech and voice perception for vocal speech signal.

### 4.3 Limitations and Future Directions

Our findings suggest that native‐language talker learning was supported primarily by higher‐level abstract representations throughout the training period. However, the relatively short duration of the study limits our ability to infer the longer‐term trajectory of learning. Within the RHT framework, enhanced sensitivity to lower‐level sensory features may emerge only after perceptual mastery has been achieved (Hochstein and Ahissar 2002; Shamma 2008; Reetzke et al. 2018). Although reliance on acoustic cues, as indexed by behavioral error patterns in this study, becomes difficult to assess once every listener's performance reaches ceiling, we would expect overtraining to increase listeners' sensitivity to lower‐level stimulus features as indexed by other measures such as frequency‐following response (e.g., Reetzke et al. 2018).

By contrast, our findings suggest that listeners relied relatively heavily on lower‐level acoustic information during non‐native‐language talker learning, and performance had not reached ceiling by the end of training. Although reliance on acoustic cues appeared to plateau in the later blocks, it remains unclear whether this pattern reflects a true asymptote or simply the limited portion of the learning curve captured within the present training window. Under an RHT account, because higher‐level abstract representations remain relatively inaccessible in the non‐native language, further improvement may increasingly depend on greater sensitivity to fine‐grained, talker‐specific acoustic detail that generalizes across sentences, thereby leading to continued reliance on lower‐level cues. An alternative possibility is that, with continued training on a restricted set of sentences, listeners would increasingly learn item‐specific acoustic patterns tied to the trained materials, rather than cues that support generalization to novel utterances. Future work that tracks pupillary responses and learning trajectories over a longer training period would help clarify how behavioral and physiological indices of learning evolve over time and how different representational levels contribute to talker learning in native and unfamiliar languages.

## 5 Conclusion

Talker identification in an unfamiliar language is less accurate than in one's native language. The pattern of greater pupil dilation response and heavier reliance on lower‐level acoustic features in unfamiliar language reveals that listeners resort to different strategies when identifying talkers in native vs. unfamiliar language: in their native language, they access the more abstract linguistic representation to identify talkers, whereas in an unfamiliar language, they have to rely on bottom‐up processing of acoustic features of talkers. Our findings are suggestive of reverse hierarchical processing of speech in talker identification, further revealing how linguistic processing and voice processing in speech perception are intertwined despite serving functionally different purposes.

## Author Contributions

**Ja Young Choi:** conceptualization, software, data curation, investigation, formal analysis, writing – original draft, writing – review and editing. **Shengyue Xiong:** data curation, investigation, formal analysis, writing – review and editing. **Jacie R. McHaney:** software, writing – review and editing. **Bharath Chandrasekaran:** funding, conceptualization, writing – review and editing.

## Funding

This work was supported by the National Science Foundation (DRL2409652).

## Conflicts of Interest

The authors declare no conflicts of interest.

## Data Availability

Data and analysis scripts are available at https://osf.io/2m8nx/?view_only=6f0fe21cf1614b1ba1338e341110eac9.

## References

[ejn70520-bib-0001] Ahissar, M. , and S. Hochstein . 2004. “The Reverse Hierarchy Theory of Visual Perceptual Learning.” Trends in Cognitive Sciences 8, no. 10: 457–464. 10.1016/j.tics.2004.08.011. 15450510

[ejn70520-bib-0002] Ahissar, M. , M. Nahum , I. Nelken , and S. Hochstein . 2009. “Reverse Hierarchies and Sensory Learning.” Philosophical Transactions of the Royal Society, B: Biological Sciences 364, no. 1515: 285–299. 10.1098/rstb.2008.0253. PMC2674477 18986968

[ejn70520-bib-0003] Aston‐Jones, G. , and J. D. Cohen . 2005. “Adaptive Gain and the Role of the Locus Coeruleus–Norepinephrine System in Optimal Performance.” Journal of Comparative Neurology 493, no. 1: 99–110. 10.1002/cne.20723. 16254995

[ejn70520-bib-0004] Barr, D. J. , R. Levy , C. Scheepers , and H. J. Tily . 2013. “Random Effects Structure for Confirmatory Hypothesis Testing: Keep It Maximal.” Journal of Memory and Language 68, no. 3: 255–278. 10.1016/j.jml.2012.11.001. PMC3881361 24403724

[ejn70520-bib-0005] Bates, D. , M. Mächler , B. Bolker , and S. Walker . 2015. “Fitting Linear Mixed‐Effects Models Using lme4.” Journal of Statistical Software 67, no. 1. 10.18637/jss.v067.i01.

[ejn70520-bib-0006] Baumann, O. , and P. Belin . 2010. “Perceptual Scaling of Voice Identity: Common Dimensions for Different Vowels and Speakers.” Psychological Research PRPF 74, no. 1: 110–120. 10.1007/s00426-008-0185-z. 19034504

[ejn70520-bib-0007] Beatty, J. 1982. “Task‐Evoked Pupillary Responses, Processing Load, and the Structure of Processing Resources.” Psychological Bulletin 91, no. 2: 276–292. 7071262

[ejn70520-bib-0008] Belin, P. , R. J. Zatorre , P. Lafaille , P. Ahad , and B. Pike . 2000. “Voice‐Selective Areas in Human Auditory Cortex.” Nature 403, no. 6767: 309–312. 10.1038/35002078. 10659849

[ejn70520-bib-0009] Berken, J. A. , V. L. Gracco , J.‐K. Chen , et al. 2015. “Neural Activation in Speech Production and Reading Aloud in Native and Non‐Native Languages.” NeuroImage 112: 208–217. 10.1016/j.neuroimage.2015.03.016. 25776210

[ejn70520-bib-0010] Boersma, P. , and D. Weenink . 2023. “Praat: Doing Phonetics by Computer.” [Computer program]. (Version 6.4.01) [Computer software]. http://www.praat.org/.

[ejn70520-bib-0011] Bogacz, R. , P. T. Hu , P. J. Holmes , and J. D. Cohen . 2010. “Do Humans Produce the Speed‐Accuracy Trade‐Off That Maximizes Reward Rate?” Quarterly Journal of Experimental Psychology 63, no. 5: 863–891. 10.1080/17470210903091643. PMC2908414 19746300

[ejn70520-bib-0012] Borghini, G. , and V. Hazan . 2018. “Listening Effort During Sentence Processing Is Increased for Non‐Native Listeners: A Pupillometry Study.” Frontiers in Neuroscience 12. 10.3389/fnins.2018.00152. PMC5859302 29593489

[ejn70520-bib-0013] Bradlow, A. R. n.d. “ALLSSTAR: Archive of L1 and L2 Scripted and Spontaneous Transcripts and Recordings.” https://speechbox.linguistics.northwestern.edu/allsstar.

[ejn70520-bib-0014] Bregman, M. R. , and S. C. Creel . 2014. “Gradient Language Dominance Affects Talker Learning.” Cognition 130, no. 1: 85–95. 10.1016/j.cognition.2013.09.010. 24211437

[ejn70520-bib-0015] Chee, M. W. , N. Hon , H. L. Lee , and C. S. Soon . 2001. “Relative Language Proficiency Modulates BOLD Signal Change When Bilinguals Perform Semantic Judgments. Blood Oxygen Level Dependent.” NeuroImage 13, no. 6 Pt 1: 1155–1163. 10.1006/nimg.2001.0781. 11352621

[ejn70520-bib-0016] Choi, J. Y. , E. R. Hu , and T. K. Perrachione . 2018. “Varying Acoustic‐Phonemic Ambiguity Reveals That Talker Normalization Is Obligatory in Speech Processing.” Attention, Perception, & Psychophysics 80, no. 3: 784–797. 10.3758/s13414-017-1395-5. PMC5840042 29417449

[ejn70520-bib-0017] de Gee, J. W. , T. Knapen , and T. H. Donner . 2014. “Decision‐Related Pupil Dilation Reflects Upcoming Choice and Individual Bias.” Proceedings of the National Academy of Sciences 111, no. 5: E618–E625. 10.1073/pnas.1317557111. PMC3918830 24449874

[ejn70520-bib-0018] Dix, A. , and S.‐C. Li . 2020. “Incentive Motivation Improves Numerosity Discrimination: Insights From Pupillometry Combined With Drift‐Diffusion Modelling.” Scientific Reports 10, no. 1: 2608. 10.1038/s41598-020-59415-3. 32054923 PMC7018719

[ejn70520-bib-0019] Fleming, D. , B. L. Giordano , R. Caldara , and P. Belin . 2014. “A Language‐Familiarity Effect for Speaker Discrimination Without Comprehension.” Proceedings of the National Academy of Sciences 111, no. 38: 13795–13798. 10.1073/pnas.1401383111. PMC4183269 25201950

[ejn70520-bib-0020] Goggin, J. P. , C. P. Thompson , G. Strube , and L. R. Simental . 1991. “The Role of Language Familiarity in Voice Identification.” Memory & Cognition 19, no. 5: 448–458. 10.3758/BF03199567. 1956306

[ejn70520-bib-0021] Gold, J. I. , and M. N. Shadlen . 2000. “Representation of a Perceptual Decision in Developing Oculomotor Commands.” Nature 404, no. 6776: 390–394. 10.1038/35006062. 10746726

[ejn70520-bib-0022] Hayes, T. R. , and A. A. Petrov . 2016. “Mapping and Correcting the Influence of Gaze Position on Pupil Size Measurements.” Behavior Research Methods 48, no. 2: 510–527. 10.3758/s13428-015-0588-x. 25953668 PMC4637269

[ejn70520-bib-0023] Hochstein, S. , and M. Ahissar . 2002. “View From the Top: Hierarchies and Reverse Hierarchies in the Visual System.” Neuron 36, no. 5: 791–804. 12467584 10.1016/s0896-6273(02)01091-7

[ejn70520-bib-0024] Jiang, X. , M. A. Chevillet , J. P. Rauschecker , and M. Riesenhuber . 2018. “Training Humans to Categorize Monkey Calls: Auditory Feature‐ and Category‐Selective Neural Tuning Changes.” Neuron 98, no. 2: 405–416.e4. 10.1016/j.neuron.2018.03.014. 29673483 PMC7371447

[ejn70520-bib-0025] Kiani, R. , T. D. Hanks , and M. N. Shadlen . 2008. “Bounded Integration in Parietal Cortex Underlies Decisions Even When Viewing Duration Is Dictated by the Environment.” Journal of Neuroscience 28, no. 12: 3017–3029. 18354005 10.1523/JNEUROSCI.4761-07.2008 PMC6670720

[ejn70520-bib-0026] Knapen, T. , J. W. De Gee , J. Brascamp , S. Nuiten , S. Hoppenbrouwers , and J. Theeuwes . 2016. “Cognitive and Ocular Factors Jointly Determine Pupil Responses Under Equiluminance.” PLoS ONE 11, no. 5: e0155574. 10.1371/journal.pone.0155574. 27191166 PMC4871560

[ejn70520-bib-0027] Koelewijn, T. , A. A. Zekveld , T. Lunner , and S. E. Kramer . 2018. “The Effect of Reward on Listening Effort as Reflected by the Pupil Dilation Response.” Hearing Research 367: 106–112. 10.1016/j.heares.2018.07.011. 30096490

[ejn70520-bib-0028] Köster, O. , and N. O. Schiller . 1997. “Different Influences of the Native Language of a Listener on Speaker Recognition.” International Journal of Speech, Language and the Law 4, no. 1: 18–28.

[ejn70520-bib-0029] Kuchinsky, S. E. , J. B. Ahlstrom , S. L. Cute , L. E. Humes , J. R. Dubno , and M. A. Eckert . 2014. “Speech‐Perception Training for Older Adults With Hearing Loss Impacts Word Recognition and Effort.” Psychophysiology 51, no. 10: 1046–1057. 10.1111/psyp.12242. 24909603 PMC4234634

[ejn70520-bib-0074] Kuchinsky, S. E. , K. I. Vaden , J. B. Ahlstrom , et al. 2016. “Task‐Related Vigilance During Word Recognition in Noise for Older Adults With Hearing Loss.” Experimental Aging Research 42, no. 1: 50–66. 10.1080/0361073x.2016.1108712. 26683041 PMC4702493

[ejn70520-bib-0030] Kuznetsova, A. , P. B. Brockhoff , and R. H. B. Christensen . 2017. “lmerTest Package: Tests in Linear Mixed Effects Models.” Journal of Statistical Software 82, no. 13. 10.18637/jss.v082.i13.

[ejn70520-bib-0031] Latinus, M. , and P. Belin . 2011. “Human Voice Perception.” Current Biology 21, no. 4: R143–R145. 10.1016/j.cub.2010.12.033. 21334289

[ejn70520-bib-0032] Leonard, M. K. , C. Torres , K. E. Travis , et al. 2011. “Language Proficiency Modulates the Recruitment of Non‐Classical Language Areas in Bilinguals.” PLoS ONE 6, no. 3: e18240. 10.1371/journal.pone.0018240. 21455315 PMC3063800

[ejn70520-bib-0072] McGarrigle, R. , P. Dawes , A. J. Stewart , S. E. Kuchinsky , and K. J. Munro . 2017. “Measuring Listening‐Related Effort and Fatigue in School‐Aged Children Using Pupillometry.” Journal of Experimental Child Psychology 161: 95–112. 10.1016/j.jecp.2017.04.006. 28505505

[ejn70520-bib-0033] McHaney, J. R. , C. L. Roark , M. J. McGinley , and B. Chandrasekaran . 2024. “Combining Pupillometry and Drift‐Diffusion Models Reveals Auditory Category Learning Dynamics.” 10.1101/2024.04.16.589753.

[ejn70520-bib-0034] McHaney, J. R. , W. L. Schuerman , M. K. Leonard , and B. Chandrasekaran . 2023. “Transcutaneous Auricular Vagus Nerve Stimulation Modulates Performance but Not Pupil Size During Nonnative Speech Category Learning.” Journal of Speech, Language, and Hearing Research 66, no. 10: 3825–3843. 10.1044/2023_JSLHR-22-00596. PMC12379271 37652065

[ejn70520-bib-0035] McHaney, J. R. , R. Tessmer , C. L. Roark , and B. Chandrasekaran . 2021. “Working Memory Relates to Individual Differences in Speech Category Learning: Insights From Computational Modeling and Pupillometry.” Brain and Language 222: 105010. 10.1016/j.bandl.2021.105010. 34454285

[ejn70520-bib-0036] McLaughlin, D. E. , Y. D. Carter , C. C. Cheng , and T. K. Perrachione . 2019. “Hierarchical Contributions of Linguistic Knowledge to Talker Identification: Phonological Versus Lexical Familiarity.” Attention, Perception & Psychophysics 81, no. 4: 1088–1107. 10.3758/s13414-019-01778-5. PMC6624079 31218598

[ejn70520-bib-0037] McMahon, C. M. , I. Boisvert , P. de Lissa , et al. 2016. “Monitoring Alpha Oscillations and Pupil Dilation Across a Performance‐Intensity Function.” Frontiers in Psychology 7. 10.3389/fpsyg.2016.00745. PMC4877370 27252671

[ejn70520-bib-0038] Meng, Y. , C. Liang , W. Chen , et al. 2024. “Neural Basis of Language Familiarity Effects on Voice Recognition: An fNIRS Study.” Cortex 176: 1–10. 10.1016/j.cortex.2024.04.007. 38723449

[ejn70520-bib-0039] Mirman, D. 2014. Growth Curve Analysis and Visualization Using R. CRC Press.

[ejn70520-bib-0040] Mullennix, J. W. , and D. B. Pisoni . 1990. “Stimulus Variability and Processing Dependencies in Speech Perception.” Perception & Psychophysics 47, no. 4: 379–390. 2345691 10.3758/bf03210878 PMC3512111

[ejn70520-bib-0041] Naber, M. , and K. Nakayama . 2013. “Pupil Responses to High‐Level Image Content.” Journal of Vision 13, no. 6: 7. 10.1167/13.6.7. 23685390

[ejn70520-bib-0042] Nahum, M. , I. Nelken , and M. Ahissar . 2008. “Low‐Level Information and High‐Level Perception: The Case of Speech in Noise.” PLoS Biology 6, no. 5: e126. 10.1371/journal.pbio.0060126. 18494561 PMC2386842

[ejn70520-bib-0043] Orena, A. J. , R. M. Theodore , and L. Polka . 2015. “Language Exposure Facilitates Talker Learning Prior to Language Comprehension, Even in Adults.” Cognition 143: 36–40. 26113447 10.1016/j.cognition.2015.06.002

[ejn70520-bib-0045] Papesh, M. H. , S. D. Goldinger , and M. C. Hout . 2012. “Memory Strength and Specificity Revealed by Pupillometry.” International Journal of Psychophysiology 83, no. 1: 56–64. 10.1016/j.ijpsycho.2011.10.002. 22019480 PMC3251658

[ejn70520-bib-0046] Paulon, G. , F. Llanos , B. Chandrasekaran , and A. Sarkar . 2021. “Bayesian Semiparametric Longitudinal Drift‐Diffusion Mixed Models for Tone Learning in Adults.” Journal of the American Statistical Association 116, no. 535: 1114–1127. 10.1080/01621459.2020.1801448. 34650315 PMC8513775

[ejn70520-bib-0047] Paulon, G. , and A. Sarkar . 2023. “lddmm: Longitudinal Drift‐Diffusion Mixed Models (LDDMM).” (R package version 0.4.0) [Computer software]. https://CRAN.R‐project.org/package=lddmm.

[ejn70520-bib-0048] Pereira, M. , P. Megevand , M. X. Tan , et al. 2021. “Evidence Accumulation Relates to Perceptual Consciousness and Monitoring.” Nature Communications 12, no. 1: 3261. 10.1038/s41467-021-23540-y PMC8166835 34059682

[ejn70520-bib-0049] Perrachione, T. K. , K. T. Furbeck , and E. J. Thurston . 2019. “Acoustic and Linguistic Factors Affecting Perceptual Dissimilarity Judgments of Voices.” Journal of the Acoustical Society of America 146, no. 5: 3384–3399. 10.1121/1.5126697. 31795676 PMC7043842

[ejn70520-bib-0071] Perrachione, T. K. , and P. C. M. Wong . 2007. “Learning To Recognize Speakers of a Non‐Native Language: Implications for the Functional Organization of Human Auditory Cortex.” Neuropsychologia 45, no. 8: 1899–1910. 10.1016/j.neuropsychologia.2006.11.015. 17258240

[ejn70520-bib-0050] Peysakhovich, V. , F. Vachon , B. R. Vallières , F. Dehais , and S. Tremblay . 2015. “Pupil Dilation and Eye Movements Can Reveal Upcoming Choice in Dynamic Decision‐Making.” Proceedings of the Human Factors and Ergonomics Society Annual Meeting 59, no. 1: 210–214. 10.1177/1541931215591043.

[ejn70520-bib-0051] Ratcliff, R. 1978. “A Theory of Memory Retrieval.” Psychological Review 85, no. 2: 59–108.

[ejn70520-bib-0052] Ratcliff, R. , and G. McKoon . 2008. “The Diffusion Decision Model: Theory and Data for Two‐Choice Decision Tasks.” Neural Computation 20, no. 4: 873–922. 18085991 10.1162/neco.2008.12-06-420 PMC2474742

[ejn70520-bib-0075] Ratcliff, R. , P. L. Smith , S. D. Brown , and G. McKoon . 2016. “Diffusion Decision Model: Current Issues and History.” Trends in Cognitive Sciences 20, no. 4: 260–281. 10.1016/j.tics.2016.01.007. 26952739 PMC4928591

[ejn70520-bib-0053] Reetzke, R. , Z. Xie , F. Llanos , and B. Chandrasekaran . 2018. “Tracing the Trajectory of Sensory Plasticity Across Different Stages of Speech Learning in Adulthood.” Current Biology 28, no. 9: 1419–1427. 29681473 10.1016/j.cub.2018.03.026 PMC5940549

[ejn70520-bib-0054] Roark, C. L. , G. Paulon , G. Rebaudo , J. R. McHaney , A. Sarkar , and B. Chandrasekaran . 2024. “Individual Differences in Working Memory Impact the Trajectory of Non‐Native Speech Category Learning.” PLoS ONE 19, no. 6: e0297917. 10.1371/journal.pone.0297917. 38857268 PMC11164376

[ejn70520-bib-0055] Roark, C. L. , G. Paulon , A. Sarkar , and B. Chandrasekaran . 2021. “Comparing Perceptual Category Learning Across Modalities in the Same Individuals.” Psychonomic Bulletin & Review 28, no. 3: 898–909. 10.3758/s13423-021-01878-0. 33532985 PMC8222058

[ejn70520-bib-0056] Serre, T. , L. Wolf , S. Bileschi , M. Riesenhuber , and T. Poggio . 2007. “Robust Object Recognition With Cortex‐Like Mechanisms.” IEEE Transactions on Pattern Analysis and Machine Intelligence 29, no. 3: 411–426. 10.1109/TPAMI.2007.56. 17224612

[ejn70520-bib-0057] Shamma, S. 2008. “On the Emergence and Awareness of Auditory Objects.” PLoS Biology 6, no. 6: e155. 18578570 10.1371/journal.pbio.0060155 PMC2435155

[ejn70520-bib-0058] Smith, P. L. , and D. Vickers . 1988. “The Accumulator Model of Two‐Choice Discrimination.” Journal of Mathematical Psychology 32, no. 2: 135–168. 10.1016/0022-2496(88)90043-0.

[ejn70520-bib-0059] Stevenage, S. V. , G. Clarke , and A. McNeill . 2012. “The “Other‐Accent” Effect in Voice Recognition.” Journal of Cognitive Psychology 24, no. 6: 647–653.

[ejn70520-bib-0060] Strauch, C. , C.‐A. Wang , W. Einhäuser , S. Van der Stigchel , and M. Naber . 2022. “Pupillometry as an Integrated Readout of Distinct Attentional Networks.” Trends in Neurosciences 45, no. 8: 635–647. 10.1016/j.tins.2022.05.003. 35662511

[ejn70520-bib-0061] Thompson, C. P. 1987. “A Language Effect in Voice Identification.” Applied Cognitive Psychology 1, no. 2: 121–131. 10.1002/acp.2350010205.

[ejn70520-bib-0062] Wierda, S. M. , H. Van Rijn , N. A. Taatgen , and S. Martens . 2012. “Pupil Dilation Deconvolution Reveals the Dynamics of Attention at High Temporal Resolution.” Proceedings of the National Academy of Sciences 109, no. 22: 8456–8460. 10.1073/pnas.1201858109. PMC3365158 22586101

[ejn70520-bib-0063] Winn, M. B. , J. R. Edwards , and R. Y. Litovsky . 2015. “The Impact of Auditory Spectral Resolution on Listening Effort Revealed by Pupil Dilation.” Ear and Hearing 36, no. 4: e153–e165. 10.1097/AUD.0000000000000145. 25654299 PMC4478109

[ejn70520-bib-0064] Winn, M. B. , D. Wendt , T. Koelewijn , and S. E. Kuchinsky . 2018. “Best Practices and Advice for Using Pupillometry to Measure Listening Effort: An Introduction for Those Who Want to Get Started.” Trends in Hearing 22: 2331216518800869. 10.1177/2331216518800869. 30261825 PMC6166306

[ejn70520-bib-0065] Xie, X. , and E. B. Myers . 2015. “General Language Ability Predicts Talker Identification.” Proceedings of the Annual Meeting of the Cognitive Science Society 37 https://escholarship.org/uc/item/17d1t53h.

[ejn70520-bib-0066] Xiong, S. , Z.‐C. Guo , G. Feng , and B. Chandrasekaran . 2025. “Dynamic Shifts in the Use of Acoustic Cues During Talker Identification: The Role of Language familiarity.” JASA Express Letters 5: 105203. 10.1121/10.0039504. 41032107

[ejn70520-bib-0067] Yu, M. E. , J. Schertz , and E. K. Johnson . 2021. “The Other Accent Effect in Talker Recognition: Now You See It, Now You Don't.” Cognitive Science 45, no. 6: e12986. 10.1111/cogs.12986. 34170043

[ejn70520-bib-0068] Zarate, J. M. , X. Tian , K. J. P. Woods , and D. Poeppel . 2015. “Multiple Levels of Linguistic and Paralinguistic Features Contribute to Voice Recognition.” Scientific Reports 5, no. 1: 11475. 10.1038/srep11475. 26088739 PMC4473599

[ejn70520-bib-0069] Zekveld, A. A. , J. M. Festen , and S. E. Kramer . 2013. “Task Difficulty Differentially Affects Two Measures of Processing Load: The Pupil Response During Sentence Processing and Delayed Cued Recall of the Sentences.” Journal of Speech, Language, and Hearing Research 56, no. 4: 1156–1165. 10.1044/1092-4388(2012/12-0058). 23785182

[ejn70520-bib-0070] Zekveld, A. A. , and S. E. Kramer . 2014. “Cognitive Processing Load Across a Wide Range of Listening Conditions: Insights From Pupillometry.” Psychophysiology 51, no. 3: 277–284. 10.1111/psyp.12151. 24506437

